# Discovery of unique mitotic mechanisms in *Paradiplonema papillatum*

**DOI:** 10.1098/rsob.250096

**Published:** 2025-08-06

**Authors:** Bungo Akiyoshi, Drahomíra Faktorová, Julius Lukeš

**Affiliations:** ^1^ Centre for Cell Biology, Institute of Cell Biology, School of Biological Sciences, University of Edinburgh, Edinburgh, UK; ^2^ Institute of Parasitology, Biology Centre, Czech Academy of Sciences, České Budějovice, Czech Republic; ^3^ Faculty of Sciences, University of South Bohemia, České Budějovice, Czech Republic

**Keywords:** kinetochore, chromosome, diplonemid, Euglenozoa, kinetoplastid

## Abstract

Diplonemids are highly diverse and abundant marine plankton with significant ecological importance. However, little is known about their biology, even in the model diplonemid *Paradiplonema papillatum* whose genome sequence is available. Examining the subcellular localization of proteins using fluorescence microscopy is a powerful approach to infer their putative function. Here, we report a plasmid-based method that enables YFP-tagging of a gene at the endogenous locus. By examining the localization of proteins whose homologs are involved in chromosome organization or segregation in other eukaryotes, we discovered several notable features in mitotically dividing *P. papillatum* cells. Cohesin is enriched on condensed interphase chromatin. During mitosis, chromosomes organize into two rings (termed mitotic rings herein) that surround the elongating nucleolus and align on a bipolar spindle. Homologs of chromosomal passenger complex components (INCENP, two Aurora kinases and KIN-A), a CLK1 kinase, meiotic chromosome axis protein SYCP2L1, spindle checkpoint protein Mad1 and microtubule regulator XMAP215 localize in between the two mitotic rings. In contrast, a Mad2 homolog localizes near basal bodies as in trypanosomes. By representing the first molecular characterization of mitotic mechanisms in *P. papillatum* and raising many questions, this study forms the foundation for dissecting mitotic mechanisms in diplonemids.

**Figure:**
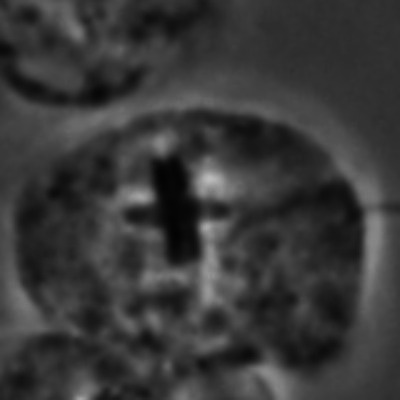

## 1. Introduction

Diplonemids are highly abundant and diverse marine microorganisms [1–3] with highly flexible life strategies [4]. They belong to Euglenozoa, an evolutionarily divergent group of flagellated eukaryotes that also includes kinetoplastids, euglenids and symbiontids [5,6]. Dissecting the biology of diplonemids is key to understanding their ecological importance as well as the origin of distinct biological processes and pathogenicity in Euglenozoa, such as trypanosomes, leishmanias and other serious pathogens. As exemplified by their membrane-trafficking machinery, the gene-rich diplonemids possess both unique and conserved proteins, proteins previously considered as kinetoplastid-specific, as well as those with sporadic distribution across eukaryotes [7].

So far, very little is known about the proliferation mechanisms in diplonemids. Classic electron microscopy studies showed that diplonemids have chromosomes that are condensed even in interphase and undergo closed mitosis with intact nucleolus [8–10]. As cells enter mitosis, the nucleolus gets elongated by an unknown mechanism. It has been reported that chromosomes form one distinct ring surrounding the elongating nucleolus, which then separates as two rings in anaphase [8,10]. Interestingly, a higher number of microtubules was observed between the separating chromosomal rings rather than between the rings and the poles [10]. Although many mitotic proteins involved in chromosome organizations in other eukaryotes are conserved in diplonemids, they have not been characterized yet. The nuclear genome of the model diplonemid *Paradiplonema papillatum* is ~280 Mb with ~32 000 protein-coding genes [11]. The size of chromosomes is 1.1−1.8 Mb, and it is estimated that there are ~180 chromosomes. It remains unknown how these numerous chromosomes organize into a ring-like structure during mitosis.

**Figure 1.**
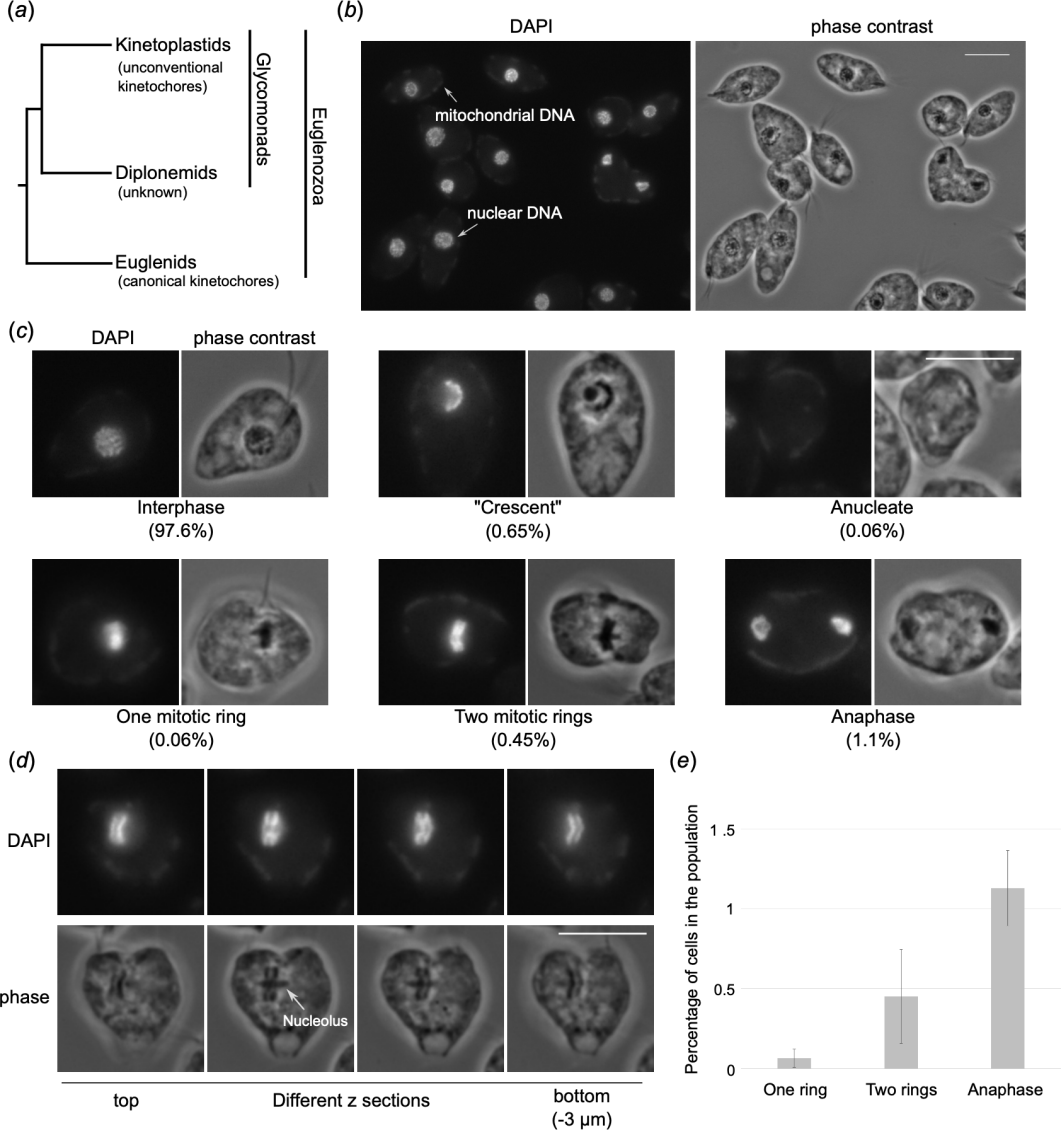
Quantification of cell cycle analysis by DAPI staining. (*a*) A current consensus Euglenozoan tree based on multiple phylogenetic analyses [16,19]. Note that euglenids have canonical kinetochore proteins, and kinetoplastids have unconventional kinetochore proteins, while it remains unknown what kind of kinetochores are present in diplonemids. (*b*) A wide field of view of *Paradiplonema papillatum* cells stained with DAPI. Scale bars, 10 µm. (*c*) Example of cells with distinct DNA morphology and their quantification. More than 1000 cells were analyzed (*n* = 3). For raw data, see electronic supplementary material, table S1. (*d*) An example of a cell that has two mitotic rings. Different z sections are shown. The raw TIFF file is included as electronic supplementary material, file S1. (*e*) Quantification of mitotic cells in the population. Error bars stand for standard deviation.

Kinetochores are the macromolecular complex that drives chromosome segregation during mitosis and meiosis in eukaryotes [12]. Although components of kinetochores are widely conserved among eukaryotes [13,14], interesting exceptions are found in Euglenozoa. While euglenids have canonical kinetochore proteins [15,16], kinetoplastids have a unique set of kinetochore proteins that are so far exclusively found in this group [17]. It remains unclear when and how the unique kinetoplastid kinetochore system evolved [18]. Because phylogenetic analysis places kinetoplastids next to diplonemids rather than euglenids [16,19] (figure 1*a*), it is important to understand diplonemid kinetochores to gain hints to this question. Interestingly, it remains unclear what kind of kinetochore proteins are present in diplonemids [16,20]. Although bioinformatics search identified homologs of some kinetoplastid kinetochore proteins, such as CLK kinases (involved in splicing) and SYCP2 and SYCP3 (involved in meiotic synapsis), these broadly conserved proteins are known to have non-kinetochore functions outside of kinetoplastids [16,20]. Using *P. papillatum* for which genome sequence and transfection methods are available [11,21], we previously found that a putative CENP-A homolog showed dot signals in interphase cells [20]. However, without any kinetochore marker available, we could not confirm that the dots represent kinetochore signals. Furthermore, due to difficulties in performing immunofluorescence microscopy in this organism, such as its poor stickiness to glass coverslips, it was not possible to determine the localization of the putative CENP-A or other proteins in mitotic cells. To overcome these issues, here we developed an endogenous YFP-tagging method and determined mitotic localization for proteins whose homologs play mitotic/meiotic roles in other eukaryotes.

## 2. Results

### 2.1. Cell cycle analysis by DAPI staining

To gain insights into the mechanism of chromosome segregation in *P. papillatum*, we first performed DAPI staining analysis on logarithmically growing cells. Due to poor stickiness to microscope glass slides or coverslips, we could not immobilize *P. papillatum* cells using a standard protocol. We therefore fixed cells in solution, resuspended them in a small volume of mounting media with DAPI and mounted cells onto a slide. This simple modification allowed us to image many cells by fluorescence microscopy (figure 1*b*). Interphase cells have chromosomes randomly distributed in the nucleus, while mitotic cells undergo chromosome alignment or separation (figure 1*c*). By imaging >1000 cells in three independently growing cultures (figure 1*c–e*; electronic supplementary material, table S1), we found that the vast majority of cells was in interphase (97.6% ± 0.5%). As observed by electron microscopy studies [8,9,22–24], chromosomes are condensed even in interphase and are visible as sausage-like structures (figure 1*c*). We also observed cells of unknown cell cycle stage with crescent-shape DNA morphology (0.65% ± 0.11%) as well as few anucleate cells (0.06% ± 0.11%). In a minority of the population (1.6% ± 0.45%), we observed cells undergoing mitosis. The following categories of mitotic stages were observed based on DNA morphology: (i) prometaphase/metaphase with one mitotic ring (0.06% ± 0.06%), (ii) ‘putative metaphase’ with two mitotic rings whose DNA intensity peaks were separated by up to 1 µm (0.45% ± 0.29%), and (iii) late anaphase when DNA is separated further and does not appear as rings (1.1% ± 0.24%). In this experiment, we did not observe cells that were in early anaphase (two rings that are separated by >1 µm but still visible as rings), because they are even rarer. In categories 1 and 2, the observed rings surrounded the elongating nucleolus (figure 1*d*), as previously reported [8,10]. Based on the fact that we observed cells with two rings much more frequently than those with one ring, we speculate that the cells with two rings are likely in metaphase, rather than early anaphase. However, if this is the case, these observations raise a number of questions: How do numerous chromosomes organize into two rings? How are duplicated chromosomes linked together (assuming that the two mitotic rings correspond to two sets of duplicated sister chromatids)? Where do kinetochores assemble on the mitotic rings? How do spindle microtubules interact with the rings? How do they achieve bi-oriented attachments?

### 2.2. C-terminal YFP-tagging method enables examination of mitotic cells in *P. papillatum*

**Figure 2.**
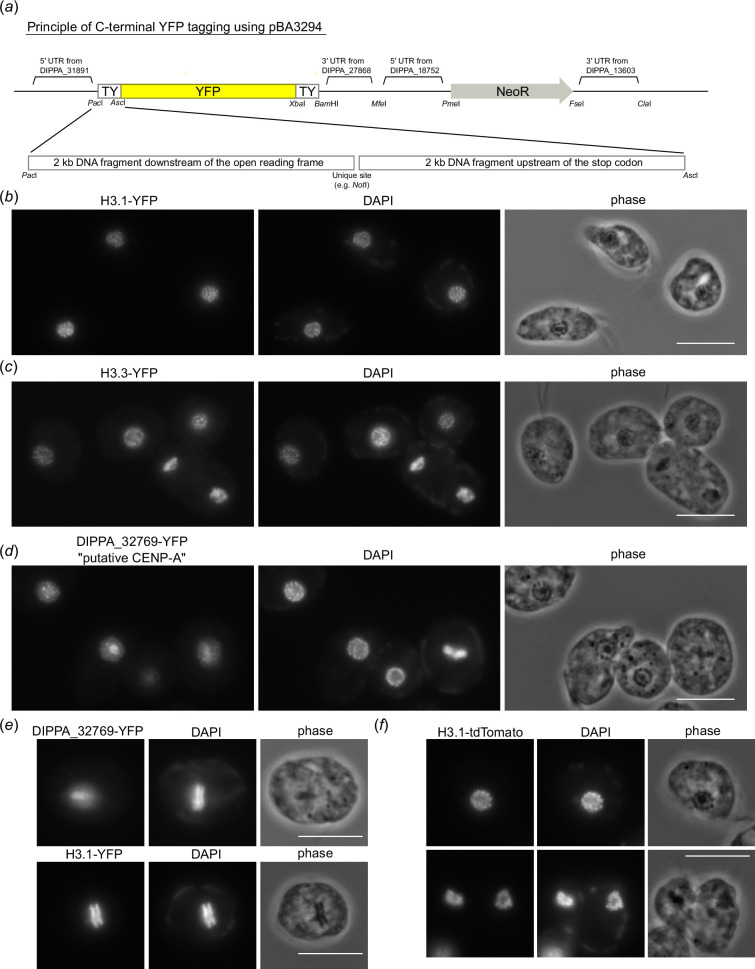
The putative CENP-A homolog does not show kinetochore localization. (*a*) Schematic of the YFP-tagging vector, pBA3294. For C-terminal tagging of a gene of interest, two homology arms are inserted into *Pac*I and *Asc*I sites of pBA3294. pBA3235 uses DIPPA_28792’s 5′UTR for YFP as well as different restriction sites. pBA3295 has tdTomato and hygromycin resistant gene instead of YFP and neomycin resistant gene of pBA3294. Full sequences of these vectors are shown in electronic supplementary material, table S2. (*b*) H3.1-YFP shows chromatin signal. Scale bars, 10 µm. (*c*) H3.3-YFP shows chromatin signal. (*d*) The putative CENP-A homolog-YFP shows dots in the nucleus. (*e*) Putative CENP-A-YFP does not localize on chromatin in metaphase, while H3.1-YFP does. (*f*) H3.1-tdTomato shows chromatin signal.

To gain insights into the above questions, we aimed to systematically examine the localization of various conserved mitotic proteins by fluorescence microscopy. In *P. papillatum*, chromosomal integration of a DNA fragment can be achieved via homologous recombination by using ~1.5 kb homology arms [21,25]. Using this method, *P. papillatum* genes have been tagged with protein A or V5 [21,26]. We adapted the system to enable YFP tagging using a plasmid-based method, similar to the one established for *Trypanosoma brucei* [27]. For C-terminal YFP tagging, two ~2 kb homology arms are amplified from genomic DNA (figure 2*a*; electronic supplementary material, table S2). The first corresponds to a 2 kb DNA fragment downstream of the open reading frame, starting just after the stop codon. The second is a fragment that starts from 2 kb upstream of the stop codon and ends just prior to the stop codon so that the open reading frame is in frame with YFP. These two DNA fragments are cloned into a vector (pBA3294 or pBA3235: YFP with a neomycin-resistant marker). A unique restriction enzyme site is introduced in between the two DNA fragments (typically *Not*I), which is used to linearize the plasmid to enable C-terminal YFP tagging of a gene at the endogenous locus. pBA3295 allows tdTomato tagging with a hygromycin selection marker. After electroporation and drug selection, a population of transgenic cells are obtained after ~10 days.

Using this method, we examined the localization of a histone H3-like protein H3.1 (DIPPA_26288) and found chromatin signal for both YFP- and tdTomato-fusion proteins (figure 2*b,f*). Another histone H3-like protein H3.3 (DIPPA_21362) also showed chromatin signal (figure 2*c*). The putative centromeric H3 variant (DIPPA_32769) has sequence features characteristic for CENP-A and forms dots in interphase cells [20]. Although dot signals were found for DIPPA_32769-YFP in interphase cells (figure 2*d*) as reported previously, the signals were not on mitotic rings during mitosis (figure 2*e*). By contrast, H3.1-YFP signals were on mitotic rings. These results do not support the possibility that DIPPA_32769 is a kinetochore protein. However, we cannot exclude a possibility that fusing YFP interfered with its proper localization.

### 2.3. Kinetoplastid-like chromosomal passenger complex compositions in *P. papillatum*

**Figure 3.**
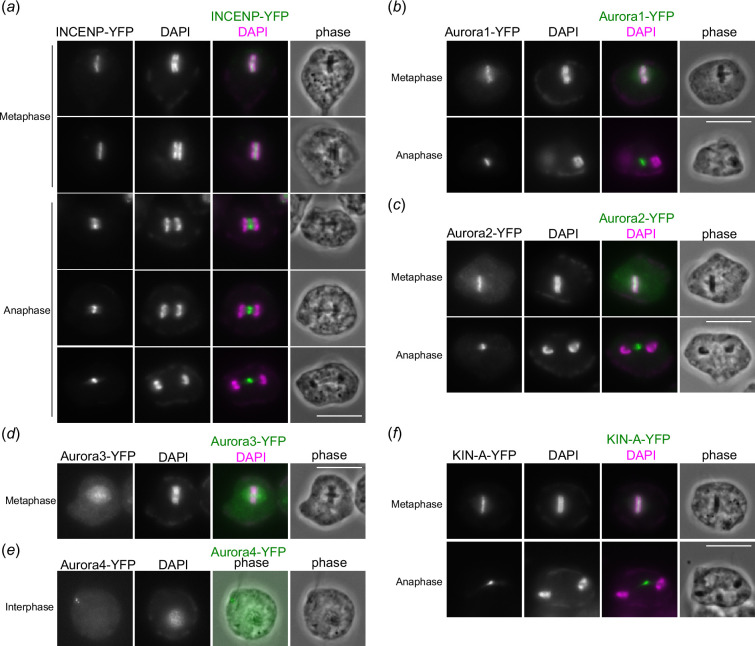
CPC in *P. papillatum* contains INCENP, two Aurora kinases and a KIN-A homolog. (*a*) INCENP-YFP shows dynamic localization pattern during mitosis. Scale bars, 10 µm. (*b*) Aurora1-YFP shows a typical CPC localization pattern. (*c*) Aurora2-YFP also shows CPC localization. (*d*) Aurora3-YFP shows a diffuse nuclear signal during metaphase. (*e*) Aurora4-YFP localizes at basal bodies. (*f*) KIN-A-YFP shows a typical CPC localization pattern.

To gain insights into mitotic mechanisms in *P. papillatum*, we next examined the localization of INCENP (DIPPA_09943), a component of the chromosomal passenger complex (CPC) that localizes near kinetochores in metaphase and then at central spindles in anaphase in many eukaryotes including kinetoplastids [28,29]. DAPI signals were used to screen rare mitotic cells. We found that *P. papillatum* INCENP localizes in between mitotic rings in metaphase and then at central spindles in early anaphase and at spindle midzone in late anaphase (figure 3*a*), thereby showing that it is a genuine CPC subunit. We next examined the localization of four Aurora homologs to identify which Aurora kinase(s) are the catalytic subunit of the *P. papillatum* CPC. Interestingly, Aurora1 (DIPPA_00804) and Aurora2 (DIPPA_18318) showed an INCENP-like localization pattern (figure 3*b,c*). In contrast, Aurora3 (DIPPA_20993) had a nuclear signal (figure 3*d*), and Aurora4 (DIPPA_24328) localized near basal bodies (figure 3*e*). These localization patterns are somewhat similar to those of *T. brucei* AUK2 that has nuclear signal [30] and AUK3 that localizes near basal bodies [31]. In addition, we found that a kinesin-like protein (DIPPA_28866) that has the highest similarity to KIN-A (a CPC subunit in *T. brucei* [32]) had a CPC-like localization pattern (figure 3*f*), raising a possibility that the CPC in *P. papillatum* is compositionally similar to that in kinetoplastids.

### 2.4. Homologs of regulatory kinetoplastid kinetochore proteins localize in between mitotic rings

**Figure 4.**
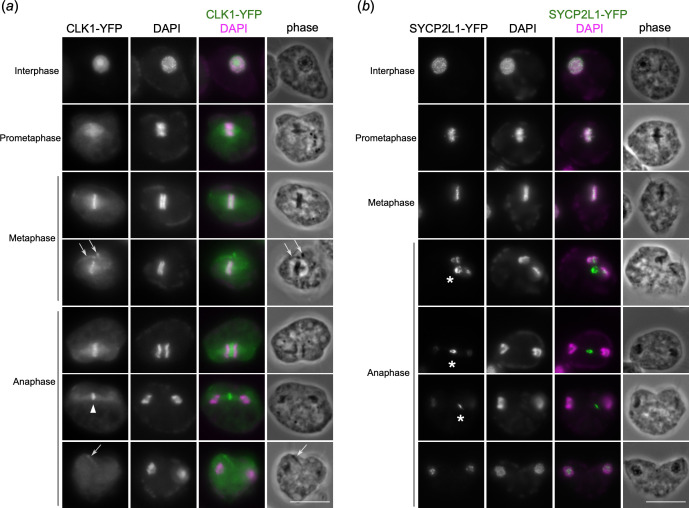
CLK1 and SYCP2 localize in between mitotic rings. (*a*) CLK1-YFP localizes in between two mitotic rings during metaphase and early anaphase, and at spindle midzone during late anaphase (arrowhead). It also has cytoskeleton signals (arrows), derived from flagella, basal bodies and/or papilla. Scale bars, 10 µm. (*b*) SYCP2L1-YFP localizes on interphase chromatin. During mitosis, the SYCP2L1-YFP signals organize into chromosomal rings during putative prometaphase and localize in between the mitotic rings. During anaphase, the signal is found in the inner side (i.e. towards the centre of the nucleus) of each ring as well as in between separated nuclei (asterisks).

We next examined the localization of broadly conserved proteins that are thus far known to have kinetochore functions only in kinetoplastids. CLK kinases regulate kinetochore functions in kinetoplastids (called KKT10 and KKT19 in *T. brucei*) [33,34], while they are involved in RNA splicing functions in other eukaryotes [35]. In *T. brucei*, KKT10 and KKT19 localize at kinetochores in metaphase and disappear in anaphase [17]. We found that CLK1 (DIPPA_05595) localized in between the mitotic rings and some cytoskeletal structures in mitotic *P. papillatum* cells (figure 4*a*). CLK1 signals are found in between the separating rings during early anaphase and at the putative spindle midzone during late anaphase.

SYCP2 homologs localize at mitotic kinetochores in kinetoplastids (called KKT17 and KKT18 in *T. brucei*), while they are used as components of the chromosome axis and synaptonemal complex during meiosis in many eukaryotes (possibly including kinetoplastids) [36,37]. We found that a SYCP2-like protein called SYCP2L1 (DIPPA_35871), which localized on chromatin in interphase, was found in between the mitotic rings (figure 4*b*), suggesting that, like KKT17 and KKT18 in *T. brucei*, SYCP2L1 may have kinetochore functions in *P. papillatum*. Besides chromatin-proximal signals, SYCP2L1 also showed an interesting signal of an unknown location in anaphase cells, which differs from CPC’s central spindle signal (figure 4*b*).

### 2.5. A divergent Mad1 homolog and XMAP215 localize in between mitotic rings

**Figure 5.**
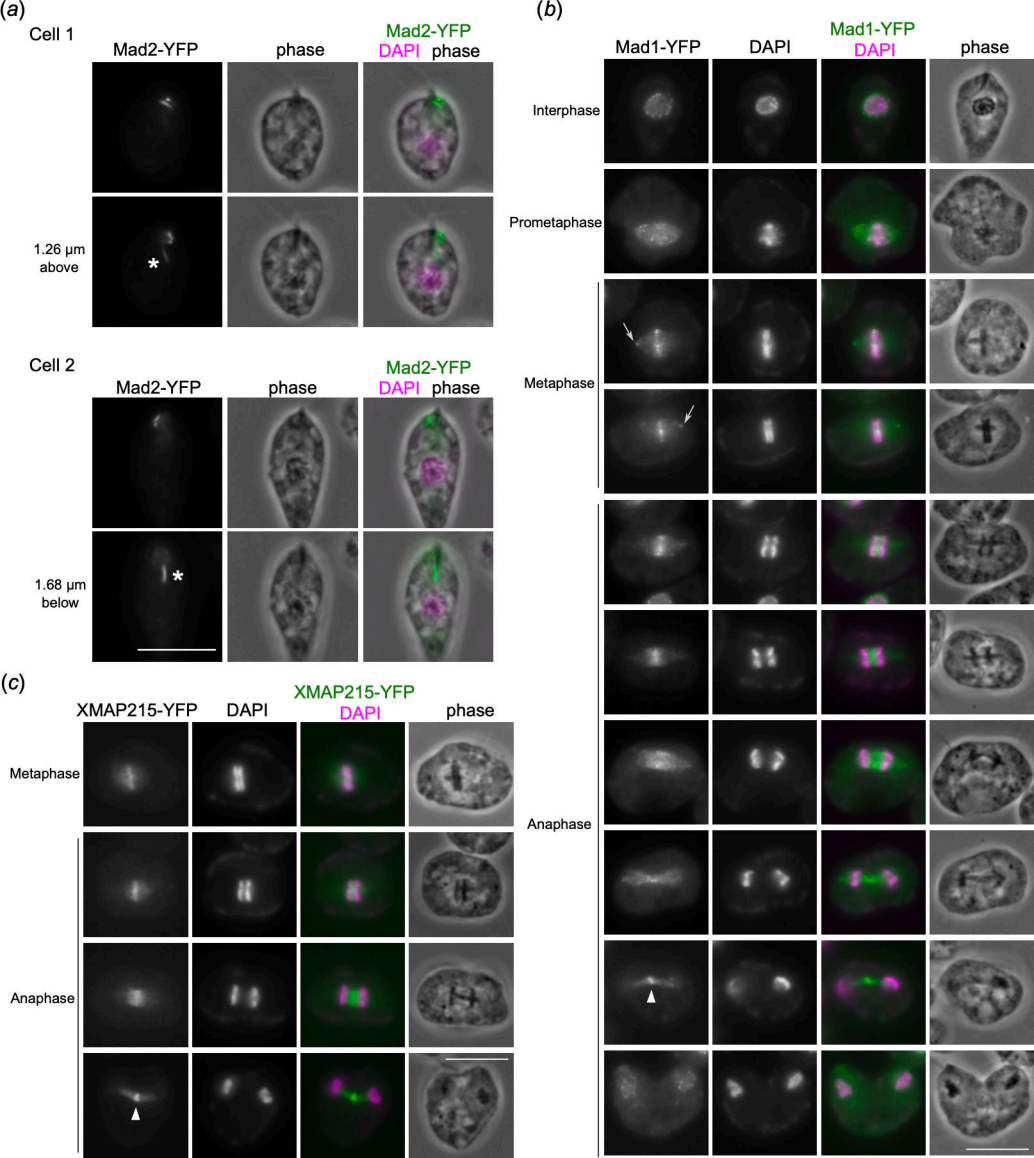
Mad1 and XMAP215 localize in between mitotic rings. (*a*) Mad2-YFP localizes near basal bodies and feeding groove (asterisks). Scale bars, 10 µm. (*b*) Mad1-YFP shows nuclear pore signals in interphase. During metaphase, the signal is found in between the mitotic rings as well as putative spindle pole areas (arrows). Mad1 localizes at the central spindle during early anaphase and at spindle midzone during late anaphase (arrowhead). (*c*) XMAP215-YFP localizes in between mitotic rings, then shows signals at the central spindle and spindle midzone (arrowhead).

We next made YFP-fusions for putative homologs of widely conserved proteins that localize at kinetochores in many organisms, but not in kinetoplastids. Spindle checkpoint proteins localize at kinetochores to monitor kinetochore-microtubule attachments [38]. We found that a spindle checkpoint Mad2 homolog (DIPPA_17925) localizes near basal bodies and feeding apparatus (figure 5*a*), the former localization pattern being reminiscent of Mad2 in *T. brucei* [39,40]. By contrast, a divergent homolog of another spindle checkpoint protein Mad1 (DIPPA_30775) localized at nuclear pores during interphase and in between mitotic rings during metaphase and early anaphase (figure 5*b*). Mad1 also showed signals at putative spindle pole areas during metaphase and at putative spindle midzone during late anaphase (figure 5*b*). A microtubule regulator XMAP215 localizes at kinetochores in metaphase and anaphase in some organisms [41,42]. We found that XMAP215 (DIPPA_00331) localized in between mitotic rings in metaphase, while central spindle and spindle midzone-like signals were observed in anaphase (figure 5*c*).

### 2.6. Robust microtubules form in between separating mitotic rings in anaphase

**Figure 6.**
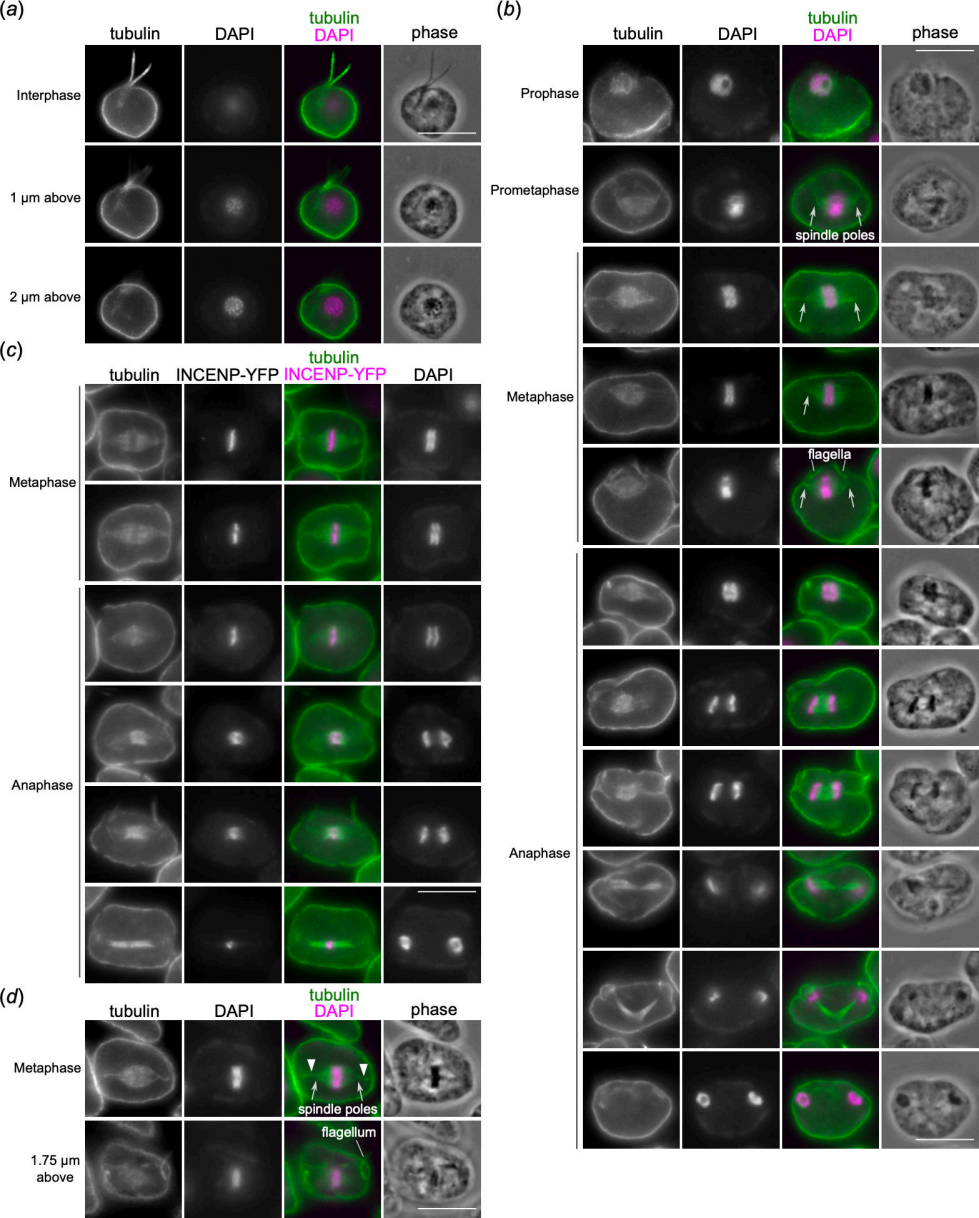
Robust microtubules form in between separating mitotic rings in anaphase. Immunofluorescence images of cells that were fixed with formaldehyde and stained with anti-tubulin antibody. Wild-type cells were used except for (*c*), which used cells expressing INCENP-YFP. Scale bars, 10 µm. (*a*) An interphase cell showing subpellicular microtubules and flagellar signals. (*b*) Nuclear signals appear in prophase. In prometaphase and metaphase, a bipolar spindle assembles in the nucleus, often showing spindle poles (arrows), which are located near the base of flagella. In early anaphase, a bipolar spindle is still visible but additional signal starts to appear in between separating rings. In mid to late anaphase, robust microtubule signals are found in between rings. (*c*) There is little overlap between INCENP-YFP and tubulin signals in metaphase. Significant overlap is visible in early anaphase. (*d*) Thin threads (arrowhead) that apparently emanate from spindle poles towards the region proximal to the base of flagella were observed in metaphase (37.5%, *n* = 32).

To examine how spindle microtubules interact with the mitotic rings, we attempted to visualize microtubules by tagging alpha and beta tubulins (DIPPA_31992 and DIPPA_16510) with YFP but failed to observe any signal. We therefore performed immunostaining of alpha tubulins (see §4). Interphase cells had strong signal in the cell periphery and flagella, but not in the nucleus (figure 6*a*). Nuclear signals were observed in mitotic cells (figure 6*b*). Prometaphase and metaphase cells formed a bipolar spindle on which mitotic rings align. Although it was difficult to precisely determine how the rings interact with spindle microtubules, we noticed signal reduction in the ring regions. Furthermore, we failed to observe enrichment of signals in between the rings during metaphase, suggesting that those proteins localized there might not interact with spindle microtubules at this mitotic stage. Indeed, little overlap was observed between tubulin and INCENP-YFP signals in metaphase cells (figure 6*c*).

In contrast, tubulin signals appeared in between the separating rings in early anaphase, when a bipolar spindle was still clearly visible. When the rings separated further, robust signal was observed in between the separating rings, which is consistent with a previous electron microscopy study in *Diplonema ambulator* that found a lot of microtubules in between the separating rings in anaphase [10]. These observations support the possibility that central spindles play an important role for anaphase chromosome movement in *P. papillatum*. In mitotic cells, spindle poles are proximal to the base of flagella. In 37.5% of metaphase cells (*n* = 32), we observed thin threads that apparently connect between spindle poles and the basal body region (figure 6*d*), which might serve as a mechanism to ensure that both the nucleus and flagella are inherited into dividing cells.

### 2.7. Cohesin localizes on interphase and mitotic chromatin, not in between mitotic rings

**Figure 7.**
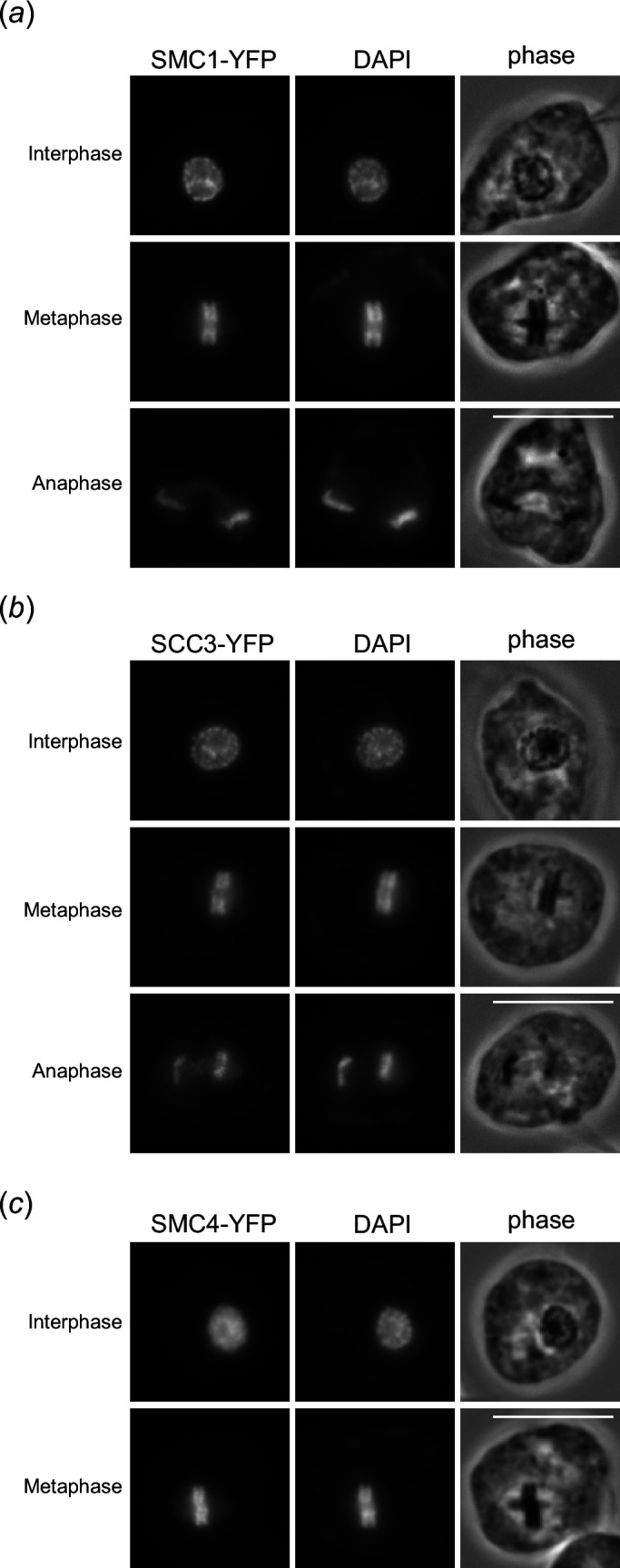
Cohesin subunits are enriched on chromatin, but not in between the mitotic rings. (*a*) A cohesin subunit SMC1-YFP shows chromatin signal in interphase, metaphase and anaphase. No obvious enrichment was observed in between mitotic rings. During anaphase, a diffuse nuclear signal was observed for SMC1 and SCC3. Scale bars, 10 µm. (*b*) SCC3-YFP shows similar localization pattern to SMC1. (*c*) A condensin subunit SMC4-YFP shows a diffuse nuclear signal in interphase and chromatin signal in metaphase.

In eukaryotes, duplicated sister chromatids are linked together by the cohesin complex [43], while the condensin complex plays major roles in chromosome organization [44]. To examine the mechanism of chromosome organization in *P. papillatum*, we examined cohesin subunits SMC1 (DIPPA_30927) and SCC3 (DIPPA_15505), as well as a condensin subunit SMC4 (DIPPA_23430). We found that cohesin subunits were enriched on interphase chromatin (figure 7*a,b*). By contrast, condensin SMC4 showed a mostly diffuse nuclear signal in interphase cells (figure 7*c*). Interestingly, both cohesin and condensin were enriched on chromatin in metaphase with no obvious enrichment in between the mitotic rings (figure 7*a–c*).

## 3. Discussion

Through YFP-tagging of genes, we have revealed several interesting features of chromosome biology in *P. papillatum*. Our finding that condensed interphase chromatin has cohesin, not condensin, suggests that cohesin may be responsible for the interphase chromosome organization in diplonemids. In mammalian cells, depletion of Wapl causes stable chromosomal association of cohesin, leading to chromatin condensation in interphase cells and chromosome mis-segregation in mitosis [45]. It will be interesting to understand how diplonemids cope with permanently condensed chromosomes.

One surprising finding from this study is the two mitotic rings with significant space in between. This raises a number of questions including how chromosomes are linked. Conservation of cohesin subunits and regulators including the Eco1 acetyltransferase (DIPPA_13679) and putative SMC3 acetylation sites (K109 and K110 in DIPPA_26840) supports the idea that sister chromatid cohesion is mediated by cohesin complexes in diplonemids, which get cleaved by separase (DIPPA_16480) at the onset of anaphase. Although we did not observe strong cohesin signals in between the mitotic rings, it is possible that a low amount of cohesin is sufficient to hold sister chromatids together. An alternative possibility is that something else holds sister chromatids together during mitosis. In female meiosis of *Bombyx mori* that lacks chiasmata, homologous chromosomes are separated by ~700 nm but are connected by a structure called the bivalent bridge that includes SYCP2, HOP1 and PCH2 [46]. It will be interesting to examine if HOP1 and PCH2 localize near mitotic rings in mitotic *P. papillatum* cells. Furthermore, we formally cannot exclude the possibility that the two mitotic rings do not correspond to duplicated sister chromatids. For example, it might be possible that one set of sister chromatids (both located within one ring) may pair up with another set of sisters (within the other ring), which could be a homologous chromosome (although *P. papillatum* is thought to be haploid [11]). However, this would mean that sister chromatids do not separate from each other during mitosis, a phenomenon never reported in eukaryotes. Further investigation is necessary to reveal the nature of mitotic rings.

The CPC localizes at metaphase kinetochores in almost all studied eukaryotes, including kinetoplastids [47]. The most conserved components of the CPC are the Aurora B kinase and its activator INCENP [14]. While recruitment of the CPC to centromeres relies on Survivin and Borealin in many eukaryotes [48], in kinetoplastids it relies on a unique component called KIN-A [32]. Our finding that the *P. papillatum* KIN-A homolog is a CPC component is consistent with the close evolutionary relationship between diplonemids and kinetoplastids (together called glycomonads) [5], which is further supported by the localization of CLK1 and SYCP2L1 on mitotic chromosomes. In this sense, chromosome segregation machinery in *P. papillatum* appears to resemble that in kinetoplastids. However, *P. papillatum* does not appear to have any homolog of structural kinetoplastid kinetochore proteins [20]. We also found that a divergent Mad1 homolog localizes in between mitotic rings not only during metaphase but also in early anaphase. The function of Mad1 remains unclear because its canonical interaction partner Mad2 localizes at different locations in *P. papillatum*. In *T. brucei*, Mad2 and its interaction partner MBP65 localize at microtubule quartet near basal bodies [31,40]. Mad2 shows a similar localization pattern in *P. papillatum*, and MBP65 homologs are present in diplonemids (DIPPA_21359 in *P. papillatum*). It will be important to dissect the function of Mad1, Mad2 and MBP65, which could provide insights into the evolutionary origin of the spindle checkpoint mechanism in eukaryotes.

Mechanisms of chromosome bi-orientation and segregation as well as the position of kinetochores in *P. papillatum* remain unknown. The CPC and XMAP215, which play key roles in error correction in other eukaryotes [41,49], localize in between mitotic rings, implying their possible roles in promoting bi-orientation in diplonemids. During anaphase, both the CPC and XMAP215 localize at central spindles. Together with the finding that there are a lot of microtubules in between the two separating rings in anaphase [10] (figure 6), these data could imply that anaphase chromosome movements are driven by the central spindle, a phenomenon observed in some eukaryotes [50–52].

In conclusion, our study starts to provide molecular insights into mechanisms of chromosome organization and segregation in *P. papillatum*. It is important to note that there is still no structural kinetochore protein identified in diplonemids, leaving the intriguing possibility open that diplonemids have a hitherto unknown type of kinetochores. In *T. brucei*, CLK1 and SYCP2 homologs co-purify with structural kinetochore components [17], while Mad1 co-purifies with kinetochore proteins in yeast [53]. It is therefore possible that immunoprecipitation coupled with mass spectrometry for these homologs could identify structural kinetochore proteins in *P. papillatum*.

## 4. Material and methods

### 4.1. Tagging vectors, plasmids and primers

Sequences of primers and tagging vectors are provided in electronic supplementary material, table S2. pBA3235 (first generation YFP-tagging vector), pBA3294 (second generation YFP-tagging vector) and pBA3295 (tdTomato-tagging vector) were synthesized by GeneArt (Thermo Fisher). For C-terminal tagging using pBA3294 (YFP) or pBA3295 (tdTomato), *Pac*I and *Asc*I restriction sites were used to insert two ~2 kb homology arms that were amplified from genomic DNA by PCR using KOD one polymerase (Merck). Primers were designed using the NEBuilder assembly tool (New England Biolabs), avoiding repetitive sequences if necessary and possible. One unique site was introduced in between the two fragments (typically *Not*I; if *Not*I was not unique, different enzymes were used): the first fragment corresponding to downstream of the open reading frame of the gene (starting just after its stop codon) surrounded with *Pac*I and *Not*I restriction sites, and the second fragment corresponding to the 2 kb DNA fragment starting from 2 kb upstream of the stop codon and ending just before the stop codon surrounded with *Not*I and *Asc*I. After cutting the fragments with respective restriction enzymes, the two DNA fragments were ligated into pBA3294 or pBA3295 that were cut with *Pac*I and *Asc*I. For C-terminal tagging using pBA3235, *Sbf*I and *Not*I restriction sites were used in combination with *Fse*I. Plasmids were screened and validated by nanopore whole plasmid sequencing (Plasmidsaurus). We occasionally detected mismatches between nanopore sequencing results and expected plasmid sequences which were made *in silico* based on the genome sequence, especially in repetitive sequences in 3′UTR regions. However, we did not attempt to figure out whether they are errors of nanopore sequencing or genome sequence.

### 4.2. Diplonema culture and transfection

All cell lines used in this study were derived from *P. papillatum* (ATCC 50162). Cells were grown at 27°C in liquid medium containing 36 g l^−1^ Instant Ocean Sea Salt (Instant Ocean), 1 g l^−1^ trypton (Formedium, TRP01) and 1% fetal bovine serum (Merck, F9665) in vented flasks.

5–10 µg plasmids were linearized by *Not*I or other enzymes, followed by ethanol precipitation. DNA was resuspended in 20 µl of transfection reagent (Ingenio Electroporation Kit for the EZporator Electroporation System, Cambridge Biosciences) and transfected into ~3 × 10^7^ cells using Amaxa Nucleofector IIb (Lonza Bioscience). Transfected cells were selected by the addition of 75 µg ml^−1^ G418 (pBA3235 and pBA3294 derivatives) or 125 µg ml^−1^ hygromycin (pBA3295 derivatives) (Merck). All cell lines used in this study are listed in electronic supplementary material, table S2.

### 4.3. Microscopy

To observe native YFP signals, 2 ml of cell culture was centrifuged at 1300*g* for 5 min. Cells were fixed by 50 µl of 4% formaldehyde solution diluted in PBS (Life Technologies, 28906) for 5 min, rinsed with 1 ml PBS twice, resuspended in a small volume (~10 µl) of DABCO mounting media (1% w/v 1,4-diazabicyclo[2.2.2]octane, 90% glycerol, 50 mM sodium phosphate pH 8.0) with 100 ng ml^−1^ DAPI, and mounted onto glass slides. For immunostaining of alpha tubulins, cells from a 20 ml culture were pelleted by centrifugation, fixed with 1 ml of 4% formaldehyde solution diluted in PBS for 5 min, and rinsed twice with 1 ml PBS (2000*g* for 3 min each), followed by incubation with the primary antibody (anti-TUB4A, T5168 clone B-5-1-2 from Merck: 1:300 dilution in 300 µl PBS) for 1 h with rotation at room temperature. Cells were rinsed twice in PBS, incubated with the secondary antibody (Alexa Fluor 488 for wild-type cells and Alexa Fluor 568 for INCENP-YFP cells, goat anti-mouse: 1:500 dilution in 300 µl PBS) for 30 min in the dark at room temperature, and rinsed twice with PBS. Cells were resuspended in mounting media and mounted onto glass slides as above. Similar results were obtained using the TAT-1 antibody [54] (1:100 dilution, overnight incubation in a cold room) (not shown). Images were captured on an Axioimager.Z2 microscope (Zeiss) installed with ZEN using a Hamamatsu ORCA-Flash4.0 camera with 63× objective lenses (1.40 NA). Typically, 15−25 z sections covering 3−6 µm were collected. Images were analyzed in ImageJ/Fiji [55]. Figures were made in Inkscape (version 1.3; https://inkscape.org/).

## Data Availability

All raw microscopy files are available upon request. Electronic supplementary material is available online [56].
